# *Porphyromonas gingivalis* activates NFκB and MAPK pathways in human oral epithelial cells

**DOI:** 10.1186/s12865-016-0185-5

**Published:** 2017-01-05

**Authors:** Sabine Groeger, Fabian Jarzina, Eugen Domann, Joerg Meyle

**Affiliations:** 1 Department of Periodontology, Justus-Liebig-University of Giessen, Giessen, Germany; 2 Institute for Medical Microbiology - German Center for Infection Research, DZIF Partner Site Giessen-Marburg-Langen - Justus-Liebig-University of Giessen, Giessen, Germany

**Keywords:** Signaling pathway, MAPK, NF-κB, Oral cells, *P. gingivalis*

## Abstract

**Background:**

The bacterial biofilm at the gingival margin induces a host immune reaction. In this local inflammation epithelial cells defend the host against bacterial challenge. *Porphyromonas gingivalis* (*P. gingivalis*), a keystone pathogen, infects epithelial cells. The aim of this study was to investigate the activation of signaling cascades in primary epithelial cells and oral cancer cell lines by a profiler PCR array.

**Results:**

After infection with *P. gingivalis* membranes the RNA﻿ of ﻿16 to 33 of 84 key genes involved in the antibacterial immune response was up-regulated, amongst them were IKBKB (NF-κB signaling pathway), IRF5 (TLR signaling) and JUN, MAP2K4, MAPK14 and MAPK8 (MAPK pathway) in SCC-25 cells and IKBKB, IRF5, JUN, MAP2K4, MAPK14 and MAPK8 in PHGK. Statistically significant up-regulation of IKBKB (4.7 ×), MAP2K4 (4.6 ×), MAPK14 (4.2 ×) and IRF5 (9.8 ×) (*p* < 0.01) was demonstrated in SCC-25 cells and IKBKB (3.1 ×), MAP2K4 (4.0 ×) MAPK 14 (3.0 ×) (*p* < 0.05), IRF5 (3.0 ×) and JUN (7.7 ×) (*p* < 0.01) were up-regulated in PHGK.

**Conclusions:**

*P. gingivalis* membrane up-regulates the expression of genes involved in downstream TLR, NFκB and MAPK signaling pathways involved in the pro-inflammatory immune response in primary and malignant oral epithelial cells.

**Electronic supplementary material:**

The online version of this article (doi:10.1186/s12865-016-0185-5) contains supplementary material, which is available to authorized users.

## Background

*Porphyromonas gingivalis* (*P. gingivalis*), an anaerobic Gram-negative rod, is a member of the oral bacterial biofilm and considered as an important etiologic agent of gingival and periodontal inflammation [1]. *P. gingivalis* is able to invade oral epithelial and endothelial cells [2–4] and effectively induces pro-inflammatory cytokine production of monocytes, neutrophils, as well as macrophages. It is also able to modify the functions of immune cells *in vitro* and *in vivo* [5, 6].

Epithelial cells not only provide a barrier against bacterial challenge and invasion but also participate in the innate immune defense. Infection of epithelial cells by *P. gingivalis* activates signaling cascades that control transcription of target genes encoding for immune response and inflammatory reactions such as interleukin (IL)-1β, IL-6, IL-8 and tumor necrosis factor (TNF)-α in monocytic and epithelial cells and interferon regulating factor (IRF) 6 in oral epithelial cells [7–9].

Pattern recognition receptors (PRRs) recognize microbial components formed as pathogen-associated molecular patterns (PAMPs). PAMPs show structural similarities between a great numbers of microorganisms, thus different PRRs usually recognize well-defined PAMPs. Toll-like receptors (TLRs) form a well-known PRR family [10]. PRRs are present on epithelial cells, neutrophils, macrophages and dendritic cells (DCs) [11]. Activation of these receptors by PAMPs initiates the innate response to microbial challenge and induces adaptive immunity to clear infections [12, 13].

Recent studies suggest that PRRs are responsible for constant surveillance of the microbial colonization by detecting conserved microbial structures such as lipopolysaccharides (LPS) [14, 15].

Intracellular invasion of pathogens is recognized by nucleotide-binding oligomerization domain (NOD)-like receptors (NLRs) which are located in the cytoplasm. Purinergic P2X receptors on the plasma membrane are activated by damaged cells [16, 17]. Ligation of the purinergic receptor, P2X7, induces the assembly of the inflammasome, a protein complex of caspase-1 and an adaptor protein ASC. Activation of caspase-1 initiates the production and release of the pro-inflammatory cytokines IL-1β and IL-18. The adaptor protein, apoptosis-associated speck-like protein NLRP3 is the best studied NLR member. It contains a CARD (ASC) domain and the protease caspase-1 [18, 19].

Gingival epithelial cells (GECs) may exhibit a functional NALP3 inflammasome. Stimulation of GECs with LPS or infection with *P. gingivalis* caused induction of the IL-1β gene and accumulation of IL-1β in the cells. However, IL-1β release did not occur unless the LPS-treated or infected cells were stimulated with adenosine triphosphate (ATP). GECs showed caspase-1 activation after treatment with ATP [20]. *P. gingivalis* expresses a nucleoside-diphosphate kinase (NDK) homolog that is able to inhibit innate immune reaction caused by stimulation with extracellular ATP. Thus, *P. gingivalis* infection inhibits ATP-induced caspase-1 activation in GECs. Furthermore *P. gingivalis* NDK may modify high- mobility group protein B1 (HMGB1) release. HMGB1 is a pro-inflammatory danger signal that, in intact cells remains associated with chromatin. HMGB1 is released into the extracellular area after stimulation of uninfected GECs with ATP instead of being translocated from the nucleus into the cytosol. In comparison to wild-type *P. gingivalis* higher amounts of HMGB1 are released when cells are infected with a NDK-deficient mutant stimulated with ATP, suggesting that NDK is crucial in inhibiting the initiation of the P2X7-dependent inflammasome and HMGB1 release from infected GECs [21].

GECs belong to the first host cells which encounter with colonizing oral bacteria. The bacterial–host communication is managed by signal transduction pathways, i.e. the mitogen-activated protein kinase (MAPK) and TLR pathway that are activated by infection with *Fusobacterium nucleatum* (*F. nucleatum*) and *Streptococcus gordonii* and other bacteria of the oral biofilm [22–24].

Molecules supporting antimicrobial clearance and the control of adaptive and innate immune responses are human beta-defensins (hBDs) produced by various cell types. Investigation of the macrophage cell line RAW 264.7 revealed that treatment with synthetic hBD3-3 peptide inhibited the LPS-induced production of inducible nitric oxide synthase and nitric oxide. Furthermore this treatment inhibited the production of secretory cytokines, such as IL-6 and tumor necrosis factor (TNF)-α in cells stimulated with LPS. This inhibition was found to be concentration-dependent. Additionally, in a model of lung inflammation, hBD3-3 was shown to reduce interstitial infiltration by neutrophils. HBD3-3 was able to downregulate the nuclear factor-kappa B (NF-κB)-dependent inflammatory response via direct suppression of the phosphorylated-nuclear factor of kappa light polypeptide gene enhancer in B-cells inhibitor alpha ﻿(IκBα) degradation and downregulation of the p65 unit of activated NF-κB [25].

*P. gingivalis* is capable of inducing immune tolerance in antigen-presenting cells (APCs) by desensitizing them against second activation, a process that involves induction of the expression of the tolerogenic molecules immunoglobulin-like transcript 3 (ILT-3) and B7-H1 [26]. It is known that T cell activation requires a co-stimulatory signal usually provided by APCs. This additional signal regulates activation or inhibition of T cell action.

In a previous study, we demonstrated that *P. gingivalis* induces B7-H1 expression in different carcinoma cell lines (SCC-25 cells, BHY cells) as well as in primary human gingival keratinocytes [27]. The B7-H1 receptor (synonymous PD-L1) belongs to the B7-family exhibiting regulatory properties that modify cell-mediated immune reactions [28, 29]. B7-H1 ligands are induced on activated T and B cells, on endothelial and epithelial cells as well as on macrophages. Dendritic cells (DCs) and APCs exhibit constitutive B7-H1expression [30–32]. The binding receptor for B7-H1 is the CD28/CTLA-4 like programmed death-1 (PD-1) receptor which is expressed on activated T cells, B cells, monocytes and macrophages. This molecule is a member of the immunoglobulin (IG) superfamily [33]. Signals mediated by B7-H1 are essential in regulating T cell activation and tolerance [34], by inhibiting functions of activated T cells. Pro-inflammatory cytokines i.e. interferon (IFN)-γ are known to up-regulate B7-H1 expression [35, 36]. Activated T cells, B cells and monocytes show PD-1 expression [37].

B7-H1 ligand binding triggers the development of regulatory T cells (T_reg_). This phenotype is essential in regulating peripheral tolerance by active suppression of effector T cells and inhibition of tissue damage caused by the inflammatory response [38–40]. Blockade of B7-H1 affected the inhibitory effect of T_reg_ [41]. Additionally, blockade of B7-H1/PD-1 ligation abolished T_reg_ mediated immune-regulation [42]. This was demonstrated in a mouse model expressing a phenotype with B7-H1 deficiency that caused diminished T_reg_ cell differentiation *in vivo* [43]. The underlying mechanisms are not completely understood. Using bladder cancer cells, B7-H1 up-regulation was shown to be induced by TLR4 signaling [44], and in oral Langerhans cells activation of TLR4 caused induction of B7-H1 *in vitro* [45, 46].

The aim of this study was to investigate the regulation of a selected number of genes after infection with *P. gingivalis*. The study was conducted to analyze mechanisms that are induced in epithelial cells after bacterial challenge. The analysis was performed on genes coding for receptor activation, downstream signal transduction, apoptosis, inflammatory response, cytokines and chemokines, and antimicrobial peptides.

## Materials and methods

### Bacteria and growth conditions

*P. gingivalis* strain W83 was purchased from the American Type Culture Collection (ATCC BAA-308™, LGC Standards GmbH, Wesel, Germany) and grown at 37 °C in brain-heart-infusion broth (Difco, BD, Heidelberg, Germany) with hemine (5 μg/ml) and menadione (1 μg/ml) (Sigma-Aldrich, Munich, Germany) under anaerobic conditions using the Anaerocult A System (Merck, Darmstadt, Germany).

### Cell cultures

The human squamous cell carcinoma cell line SCC-25 was purchased from the DSMZ (German Collection of Microorganisms and Cell Cultures, Braunschweig, Germany, DSMZ number ACC 617) and cultured in a medium containing Dulbecco’s minimal essential medium (DMEM):Ham’s F12 (1:1, vol:vol), (Invitrogen, Karlsruhe, Germany) and 20% fetal calf serum (FCS, Greiner, Frickenhausen, Germany). Primary human gingival keratinocytes (PHGK) were obtained from gingival biopsies of healthy volunteers, prepared and cultured in a serum-free medium containing DMEM:Ham’s F12 (4:1, vol:vol), 10 mM HEPES (Invitrogen, Karlsruhe, Germany).

### Bacterial cell fractionation

The bacteria were harvested in the late exponential growth phase (OD_600_ of 1.0) by centrifugation for 20 min at 6,500 × *g* and 25 °C. The bacterial pellet was re-suspended in 50 ml of 10 mM HEPES, pH 7.4, containing protease inhibitor cocktail (4 mini-tablets of Complete, EDTA-free, Roche) and DNase I/RNase A (20 μg/ml each). Bacteria were disrupted by four passages through a high-pressure cell disruption system (Model TS, 0.75 KW, Constant Systems Ltd.) at 40,000 psi. The cellular debris was removed by centrifugation at 8,000 × *g* for 30 min at 4 °C, and the membranes were sedimented from the cleared lysate at 150,000 × *g* for 2 h at 4 °C. The supernatant (cytosolic fraction) was stored, and the total membrane fraction was washed three times with 10 mM HEPES, pH 7.4. The membrane pellet was subsequently re-suspended in 10 mM HEPES, pH 7.4. The protein concentrations of all samples, i.e. cleared lysate, cytosolic fraction and total membranes, were determined using Bio-Rad’s protein assay reagent. The purity of the fractions was confirmed by sodium dodecyl sulfate polyacrylamide gel electrophoresis (SDS PAGE) using a 10% gel following staining with coomassie brilliant blue (SERVA Electrophoresis GmbH, Heidelberg, Germany).

### Infection of SCC-25 cells and membrane-stimulation of SCC-25 cells and PHGK

For infection of SCC-25 cells and primary human gingival keratinocytes (PHGK), the cells were seeded in 6-well plates (1×10^6^ cells/well) in antibiotic-free medium containing 1.8 mM calcium chloride and 10% FCS (Thermo Fisher Scientific, Darmstadt, Germany) and grown at 37 °C in a humidified atmosphere with 5% CO2 to 80% confluency before stimulation.

Cells were infected with whole bacterial cells as well as treated with bacterial fractions. To prepare *P. gingivalis* W83 for infection, the bacterial cells were harvested in the late exponential growth phase (OD_600_ of 1.0) by centrifugation at 25 °C for 20 min at 6,500 × *g*. The supernatant was discarded, and the cell pellet was resuspended in DMEM:Ham’s with 10% FCS, adjusting the bacterial cell number on the basis of spectrophotometric measurements of the optical density of the bacterial suspension at 600 nm (OD_1_ = 10^9^ cells/ml). Infection of the SCC-25 cells was performed at a multiplicity of infection (MOI) of 100 for 24 h. The bacterial membrane fractions from *P. gingivalis* W83 was used in a concentration of 50 μg/ml. A non-treated control containing cells only in culture medium was carried in every experiment. SCC-25 cells and PHGK were treated with the bacterial fractions for 24 h at 37 °C, 95% air, 5% CO_2_ and 92% relative humidity and harvested by scraping in RNA protect solution (Qiagen) for RNA extraction. All analyses were performed in three independent experiments.

### RNA extraction

Total RNA was extracted using RNeasy mini columns with on-column DNase treatment following the manufacturer’s instructions (Qiagen). The concentration and quality of the RNA were analysed using a NanoDrop 2000 spectrophotometer (Thermo Fisher Scientific, Darmstadt, Germany). The integrity of the RNA was verified using RNA gel electrophoresis.

### Human Antibacterial Response RT^2^ Profiler Array

The Human Antibacterial Response RT^2^ Profiler PCR Arra/cat. No. 330231 PAHS-148Z (Qiagen, Hilden, Germany) was used to profile the expression of 84 key gens involved in innate immune response to bacteria.

Synthesis of the cDNA was performed with the RT^2^ first strand kit (Qiagen) according to the manufacturer’s instructions at 42 °C for 15 min with a 5-min deactivation step at 95 °C in an BioRad CFX96 Real-Time System C1000 Thermal Cycler (Biorad, Munic, Germany).

The RT^2^ SYBR green master mix (Qiagen) (1350 μl per 96-well plate) was mixed with 1248 μl RNase free water and 102 μl cDNA synthesis reaction template, and 25 μl PCR components were added to each well of the array. Quantitative real time polymerase chain reaction (qRT-PCR) was performed in accordance with the recommendations of the manufacturer. Cycling and detection were done in a Bio Rad CFX96 real time system C1000 thermal cycler (Bio Rad).

### qRT-PCR for verification of profiling

Synthesis of cDNA was performed using the Verso™ cDNA Kit (Thermo Fisher Scientific) following the manufacturer’s instructions. qRT-PCR using the SYBR Green Assay was performed with SensiFast no ROX SYBR Green Mix (Bioline, Luckenwalde, Germany) according to the manufacturer’s recommendations. The following primers were used: QuantiTect Primer Assay (Qiagen) Hs_NFKB1_1_SG (NF-κB1), HS_IKBKB_1_SG (IKKβ), Hs_MAP2K4_1_SG (MAP2K4), Hs_MAPK8_1_SG (MAPK8), Hs_MAPK14_1_SG (MAPK14), Hs_IRF5_1_SG (IRF5), Hs_JUN_1_SG (Jun), Hs_IRAK3_1_SG (IRAK3), Hs_TOLLIP_1_SG (TOLLIP), and Hs-GAPDH_1_SG (GAPDH) as a housekeeping gene (patents: Roche Molecular Systems). Cycling and detection was performed in a Biorad CX96 cycler (Biorad, Munic, Germany). All samples were tested 3 × in triplicate (*n* = 9).

### Data analysis

The analysis of the profiler arrays was performed using the online analysis tool of the manufacturer based on changes in gene expression for pair-wise comparison with the non-treated control using the ∆∆Ct method. The results of the qRT-PCR were analyzed using the comparative CT (ΔΔCT) method. The amount of target (2^-ΔΔCT^) was obtained by normalizing to an endogenous reference (GAPDH) relative to non-infected control cells. The results are shown as log2 fold (x) regulation.

### Statistical analysis

The results were analyzed using independent two-sample Student’s *t*-test. The character of the evaluation was explorative. Probability of error was set to 5% and shown as *p*-values.

## Results

### SCC-25 cells treated with *P. gingivalis* W83 isolated membrane

The analysis of three experiments treating SCC-25 cells with the membrane fraction for 24 h showed up-regulation of a number of genes that play a role in different biological processes. Up-regulated were genes involved in the TLR signaling cascade, in the NF-κB pathway and the MAPK pathways. Statistically significant with a *p*- value of < 0.05 was the up-regulation of *IBKB* (4.0 ×) and *JUN* (8.7 ×). The results of this analysis are shown in Table 1. The Ct values are shown in Additional file 1: Table S4.

**Table 1:** Up-regulated genes in SCC-25 cells after stimulation with the membrane fraction of *P. gingivalis* W83.

Gene Symbol	Fold Regulation	Biological Function
IRAK1	2.5	TLR Signaling
IRAK3	2.2	
IRF5	3.1	
TICAM1	2.6	
TOLLIP	2.3	
TRAF6	2.8	
HSP90AA1	2.0	NLR Signaling
IKBKB*	4.0	NF-κB Pathway
NFKB1	2.6	
RELA	2.5	
Jun*	8.7	MAPK Pathway
MAP2K1	1.5	
MAP2K4	2.9	
MAPK1	2.6	
MAPK14	2.9	
MAPK8	2.4	

Mean values from 3 experiments as x-fold regulation compared to the non-infected control. * = *p* < 0.05

### SCC-25 cells infected with *P. gingivalis* W83 living bacteria

Infection of SCC-25 cells with *P. gingivalis* W83 for 24 h induced up-regulation of genes also with biological functions in TLR signaling, the NF-κB pathway and MAPK downstream pathway, as well as the cytokine IL-*12A*. Statistically significant (*p* < 0.05) was the up-regulation of *IKBKB* (3.1 ×) *MAP2K4* (2.7 ×), *MAPK14* (2.7 ×) and *MAPK8* (2.6 ×). The results of this analysis are shown in Table 2. The Ct values are shown in Additional file 1: Table S4.

**Table 2:** Up-regulated genes in SCC-25 cells after stimulation for 24 h with living *P. gingivalis* W 83.

Gene Symbol	Fold Regulation	Biological Function
IRAK3	2.8	TLR Signaling
IRF5	2.7	
RAC1	3.0	
TICAM1	2.5	
TOLLIP	2.2	
RELA	2.7	
CASP8	2.8	NLR signaling, Apoptosis
IKBKB *	3.1	NF-κB Pathway
NFKB1	3.6	
JUN	3.2	MAPK Pathway
MAP2K1	3.6	
MAP2K4 *	2.7	
MAPK1	3.3	
MAPK14 *	2.7	
MAPK8 *	2.6	
CCL5	2.2	Chemokines
IL12A	2.4	Cytokines
CASP1	4.9	Apoptosis

Mean values from 3 experiments as x-fold regulation compared to the non-infected control. * = *p* < 0.05

### PHGK stimulated with *P. gingivalis* W83 membrane

The analysis of three experiments treating PHGK cells with membrane fraction for 24 h showed up-regulation of various genes as well. Up-regulated were genes that participate in the TLR signaling, in NLR signaling, apoptosis, inflammatory processes, the NF-κB pathway and the MAPK downstream signaling. Further up-regulated genes were related to inflammatory response, chemokines, apoptosis and antimicrobial peptides. Also *DMBT1,* a tumor suppressor gene that participates in various biological processes like mucosal immune response, was up-regulated. The up-regulation of *IRF5* was significant (*p* < 0.05, 14.3 ×). The results of this analysis are shown in Table 3. The Ct values are shown in (Additional file 2: Table S5).

**Table 3:** Up-regulated genes in primary human gingival keratinocytes 24 h of infection with membrane fractions of *P. gingivalis* W83.

Gene Symbol	Fold Regulation	Biological Function
IRAK3	2.5	TLR Signaling
IRF5*	14.3	
TRAF6	2.6	
HSP90AA1	2.7	NLR Signaling
NOD1	2.3	
NOD2	2.0	
XIAP	2.4	
NAIP	2.3	
BIRC3	2.2	NLR Sign., Apoptosis
IKBKB	4.0	NF-κB Pathway
NFKB1	2.6	
NFKBIA	2.4	
MAP2K4	2.6	MAPK Signaling
MAPK14	2.3	
MAPK8	2.0	
CRP	3.8	Inflammatory Response
LBP	2.6	
LY96	2.6	
AKT1	2.1	Inflam. Resp., Apoptosis
CCL3	3.2	Chemokines
CCL5	3.0	
CXCL2	2.1	
IL-12A	2.2	Cytokines
IL-12B	3.3	
IL18	2.7	
CASP1	2.1	Apoptosis
PYCARD	3.5	
RIPK1	3.3	
BPI	3.2	Antimicrobial Peptides
CAMP	3.3	
MPO	2.3	
SLPI	3.6	
DMBT1	4.0	Mucosal Immune Response

Mean values from 3 experiments as x-fold regulation compared to the non-infected control. * = *p* < 0.05

### Quantitative real time polymerase chain reaction (PCR)

**Fig. 1:**
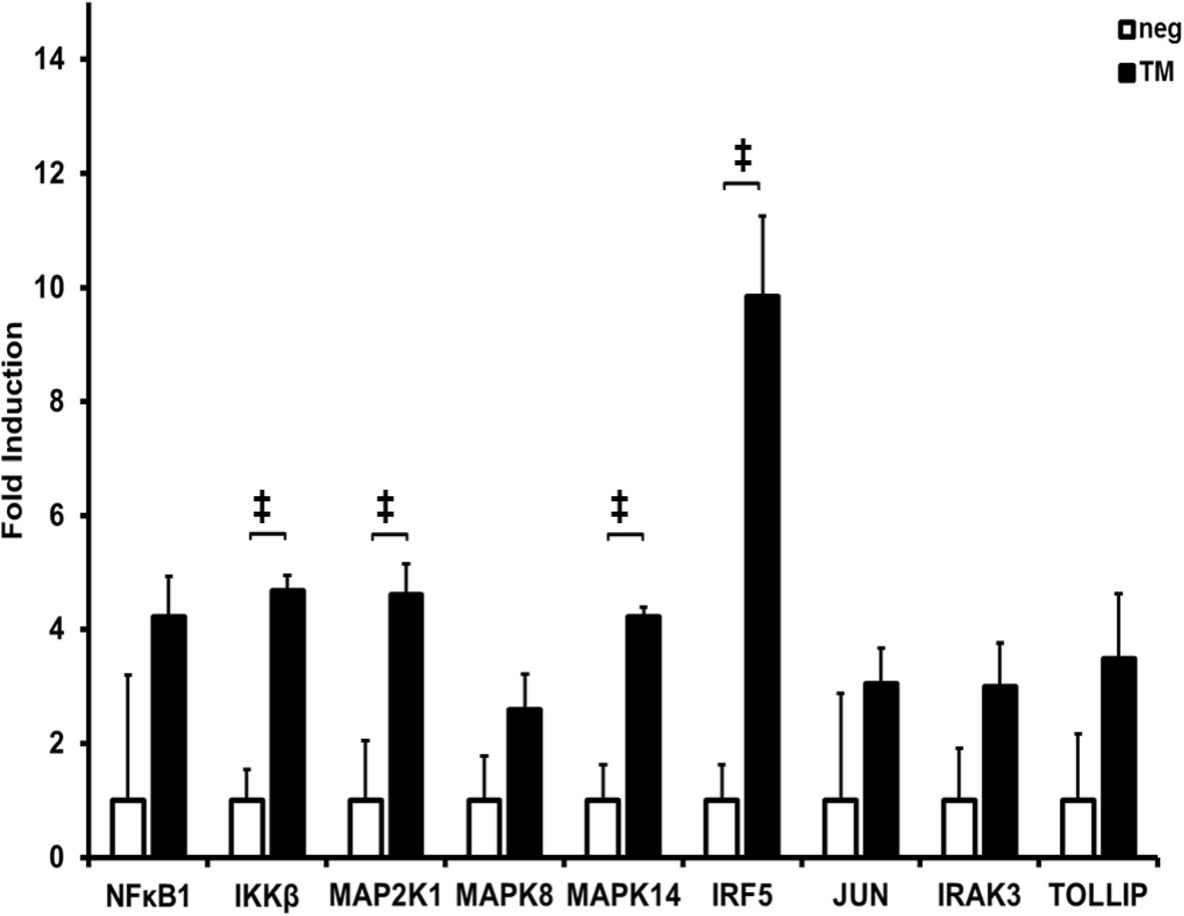
Up-regulation of genes in *P. gingivalis* membrane stimulated SCC-25 cells. Up-regulation of NF-κB, IKBKB, MAP2K4, MAPK8, MAPK 14, IRF5, JUN, IRAK3 and TOLLIP in SCC-25 cells after 24 h stimulation with *P. gingivalis* total membrane fraction (= TM) analyzed by ΔΔCt method, shown as absolute fold induction of RNA expression relative to non-stimulated samples as negative control (= neg), normalized to the house keeping gene GAPDH, *n* = 9, ‡ = *p* < 0.01

Quantitative real time PCR (qRT-PCR) of RNA in SCC-25 cells after 24 h of infection with *P. gingivalis* total membrane (Fig. 1) showed statistically significant up-regulation of the following genes: *IκBκB* (4.7 ×), *MAP2K4* (4.6 ×), *MAPK14* (4.2 ×) and *IRF5* (9.8 ×) (*p* < 0.01) (*n* = 9). Slightly up-regulated were *NFκB1* (4.4 ×), *MAPK8* (2.6 ×), *JUN* (3 ×), *IRAK3* (3.0 ×) and *TOLLIP* (3.5 ×) (*p* > 0.05).

**Fig. 2:**
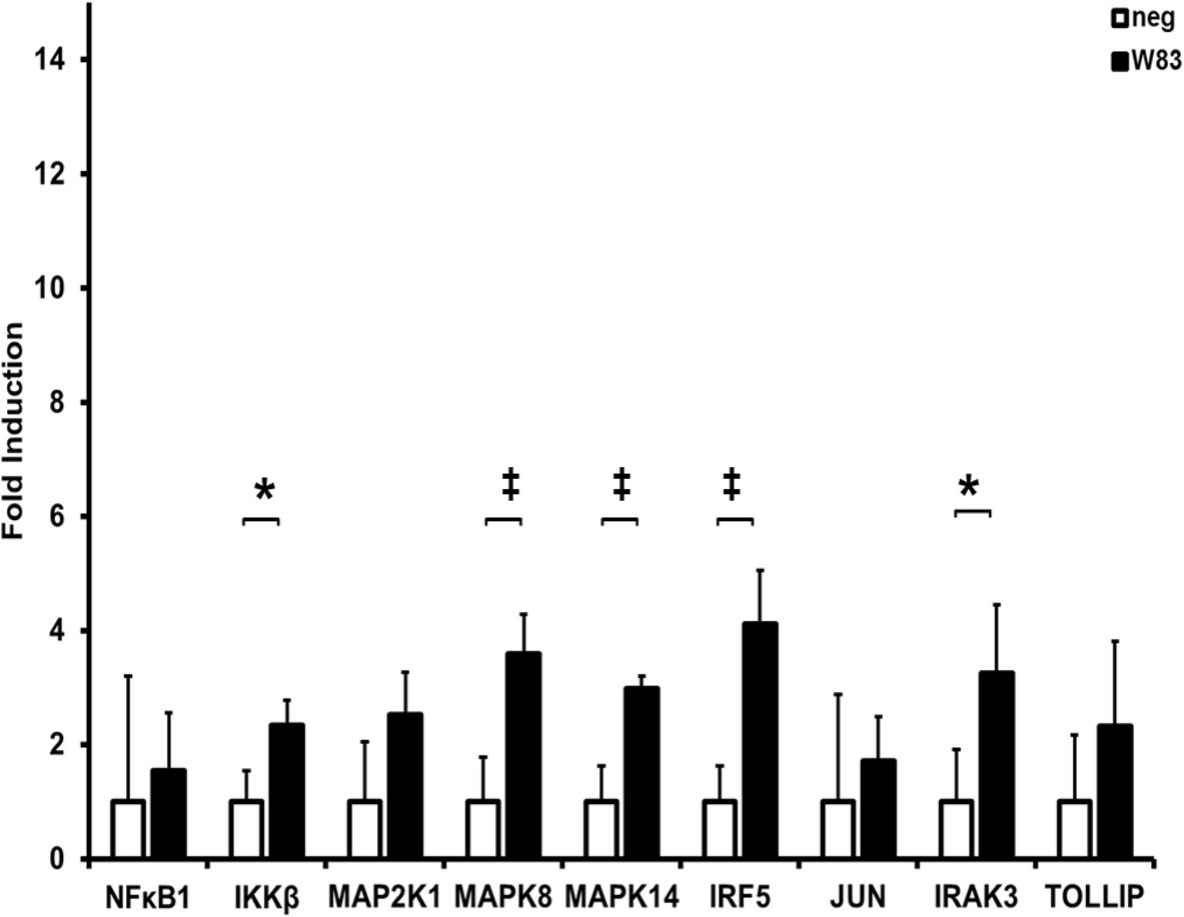
Up-regulation of genes in *P. gingivalis* bacteria stimulated SCC-25 cells. Up-regulation of NF-κB, IKBKB, MAP2K4, MAPK8, MAPK 14, IRF5, JUN, IRAK3 and TOLLIP in SCC-25 cells after 24 h stimulation with *P. gingivalis* W83, whole bacteria (= W83), analyzed by ΔΔCt method, shown as absolute fold induction of RNA expression relative to non-stimulated samples as negative control (= neg), normalized to the house keeping gene GAPDH, *n* = 9, * = *p* < 0.05, ‡ = *p* < 0.01

Real-time RNA quantification of SCC-25 cells upon stimulation *P. gingivalis* whole bacteria (Fig. 2) showed statistically significant up-regulation of *IκBκB* (2.3 ×), *IRAK3* (3.3 ×) (*p* < 0.05), *IRF5* (4.1 ×), *MAPK8* (3.6 ×) and *MAPK* 14 (3.0 ×) (*p* < 0.01) (*n* = 9). Only slightly up-regulated were *NFκB1* (1.5 ×), *MAP2K4* (2.5 ×), *JUN* (1.7 ×) and *TOLLIP* (2.3 ×).

**Fig. 3:**
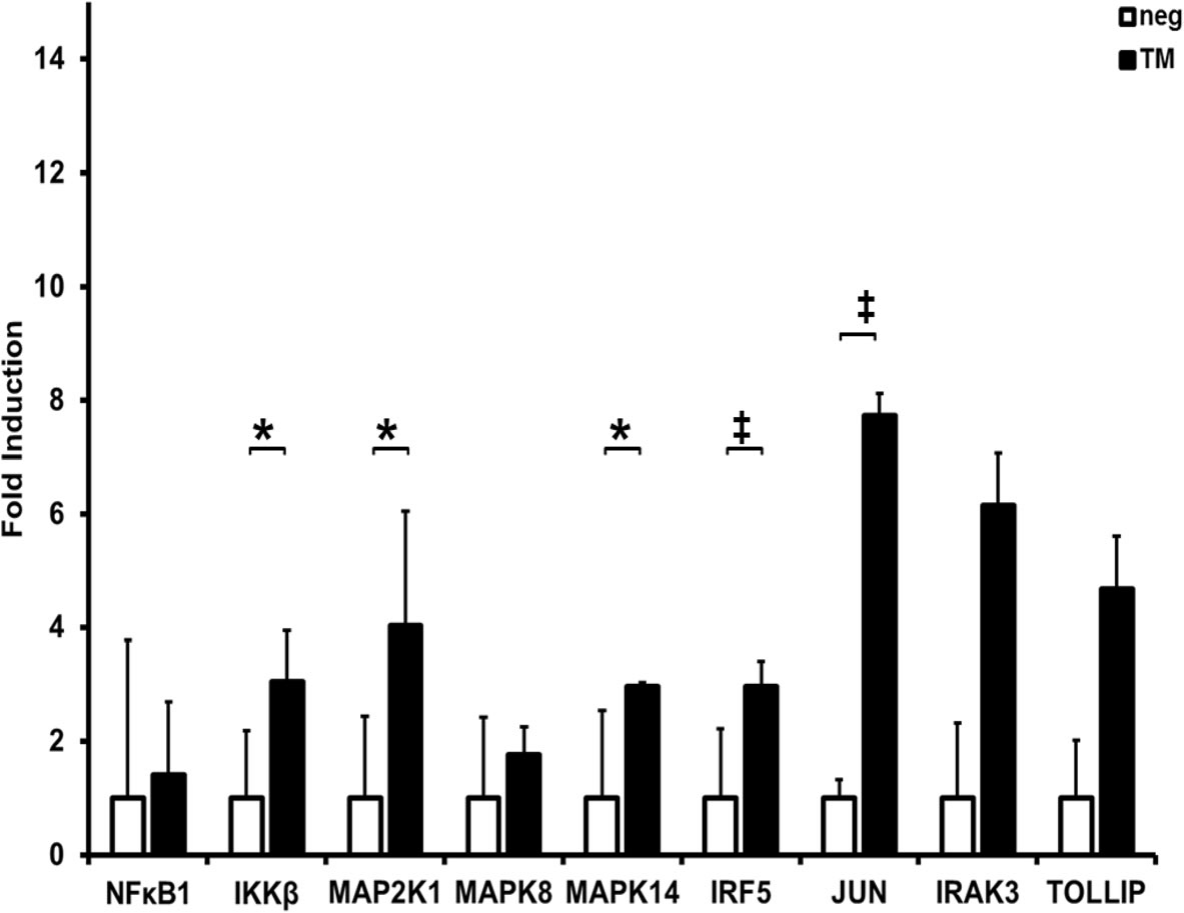
Up-regulation of genes in *P. gingivalis* membrane stimulated PHGK. Up-regulation of NF-κB, IKBKB, MAP2K4, MAPK8, MAPK 14, IRF5, JUN, IRAK3 and TOLLIP in PHGK cells after 24 h stimulation with *P. gingivalis* total membrane fraction (= TM), analyzed by ΔΔCt method, shown as absolute fold induction of RNA expression relative to non-stimulated samples as negative control (= neg), normalized to the house keeping gene GAPDH, *n* = 9, * = *p* < 0.05, ‡ = *p* < 0.01

In primary human epithelial cells stimulation with *P. gingivalis* total membrane (Fig. 3) resulted in up-regulation of *IκBκB* (3.1 ×), *MAP2K4* (4.0 ×) *MAPK* 14 (3.0 ×) (*p* < 0.05), *IRF5* (3.0 ×) and JUN (7.7 ×) (*p* < 0.01) (*n* = 9). *NFκB1* (1.4 ×), *MAPK8* (1.8 ×), *IRAK3* (6.1 ×) and *TOLLIP* (4.7 ×) were also up-regulated as well (*n* = 9) (*p* > 0.05).

**Fig. 4:**
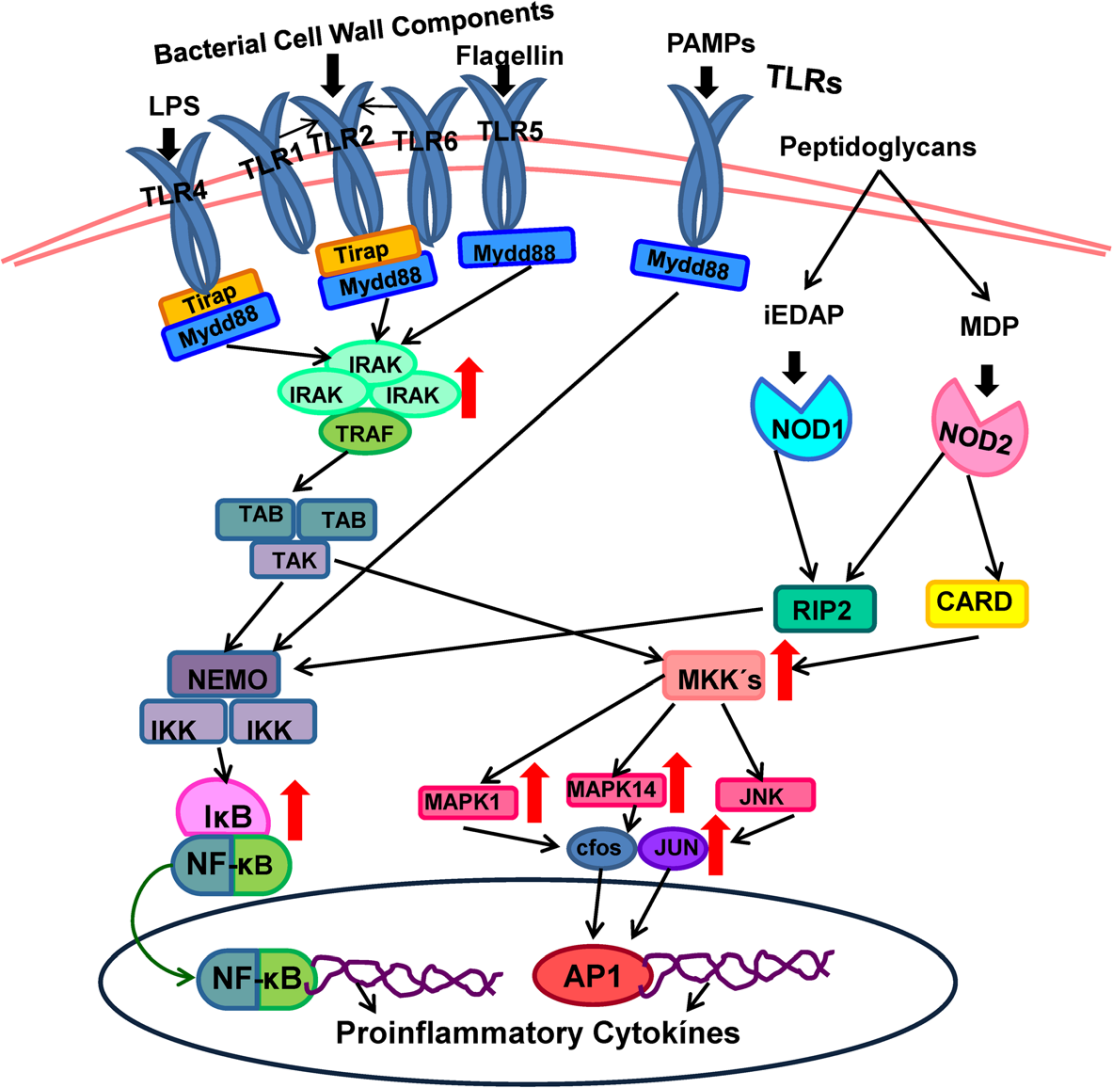
Pathogen associated pattern recognition receptor activated signaling pathways. Graphics of the nuclear factor-kappa B (NF-κB) and mitogen- activated protein kinase (MAPK or MKK) signaling pathways induced by activation of pathogen associated pattern recognition receptor (PAR) toll like receptors (TLR) and nucleotide-binding oligomerization domain receptors (NOD). TLRs and NODs belong to the key initiators of inflammation in host defence. Diffferent TLRs recognize differencial microbial components. TLR4 detects lipopolysaccharide (LPS), TLR1/2 and TLR2/6 recognize triacylated and diacylated lipoproteins from bacterial wall components and TLR5 is activated by flagellin from the flagella of multiple bacteria. TLRs signal via the adaptor protein MyD88, leading to transforming growth factor-β-activated kinase 1 (TAK1) activation that induces NF-kB and p38/c-Jun N-terminal kinase (JNK) pathways. Recognition of NOD ligands recruit caspase activation and recruitment domain (CARD) interaction with receptor-interacting protein kinase RIP2 which leads to activation of RIP2. RIP2 mediates activation IκB kinase. The activation of IκB kinase results in the phosphorylation of inhibitor IκB which releases NF-κB and its nuclear translocation. NF-κB and p38/JNK activated activator protein 1 (AP-1) function as transcription factor for the expression of inflammatory cytokines. The red arrows indicate the upregulated genes in oral epithelial cells induced by *P. gingivalis* and its total membrane that were detected in this study

Fig. 4 shows the nuclear- factor kappa B (NF-κB) and mitogen activated protein kinase (MAPK or MKK) signaling pathways induced by activation of toll-like receptors (TLRs) and nucleotide-binding oligomerization domain receptors (NODs). Upregulated genes in oral epithelial cells induced by *P. gingivalis* and its total membrane are indicated by red arrows.

## Discussion

Periodontitis is mainly caused by an oral microbial biofilm, however, progression of the disease is regulated by the immune-inflammatory reaction and the destruction of the teeth supporting tissues [47]. *P. gingivalis* plays an essential role in the pathogenesis and progression of periodontitis. Among many different mechanisms, it has been shown that *P. gingivalis* differentially activates the NF-κB pathway. After infection with *F. nucleatum* the oral epithelial cell line H400 responded with activation of NFκB. However, a significantly higher number of NF-kB translocations into the nucleus were detected after H400 cell infection with *F. nucleatum* suggesting that these two periodontal pathogens have different molecular influences on these cells [48]. In human monocyte-derived macrophages, *P. gingivalis* gingipains induced secretion of TNF-α and IL-8 and upon stimulation the amount of phosphorylated p38α MAPK increased [49].

Upon stimulation of primary oral epithelial cells and carcinoma cells with bacterial fractions of *P. gingivalis*, a number of genes were conjointly up-regulated. The genes *IKBKB*, *IRAK3*, *IRF5*, *MAP2K4* (MEK4), *MAPK14* (p38), *MAPK8* (JNK1) and *NFKB1* (p50) were upregulated not only in both cell types, but also after infection with whole bacteria of *P. gingivalis* W83 as well as with the membrane fraction. The cytosolic fraction didn’t induce altered gene expression (data not shown). NF-KB1 (p50) is a protein subunit of the NF-κB protein complex, a transcription factor central for a number of immunological and inflammatory reactions, including five subunit members – RelA (p65), RelB, c-Rel, p50 (NF-KB1) and p52 with functions as homodimers and heterodimers [50]. The NF-κB transcription factors are dissociated in the cytoplasm by a family of inhibitors of κB, the IκBs. The IκB kinase (IKK) complex, including IKBKB, initiates the activation and is activated as well. Further phosphorylation and disintegration of IκB protein results in activation of NF-κB [51]. Mitogen-activated protein kinases (MAPKs) are a highly conserved family of Ser/Thr protein kinases in eukaryotes that are regulating a number of cellular activities such as managing cellular responses to cell stress, and pro-inflammatory cytokines.

Epithelial cells, such as GECs, are able to respond to bacterial challenge by initiation of a deliberated signaling network. The GECs express different receptors on the cell surface or in the cytoplasm. Their activation induces innate immune reactions, including TLRs, nucleotide binding oligomerization domain receptors (NODs) and protease-activated receptors (PARs). It has been shown that surface receptors, such as TLRs and PARs, are activated when corresponding bacterial motifs or proteases are detected. Thus, activation of TLRs and PARs leads to downstream activation of NF-κB and/or MAPK pathways [52–55]. Activations of TLR and PARs by membrane fractions induce the up-regulation of downstream signaling molecules that were detected in this study. The TLR family shares downstream signaling molecules, amongst them the adaptor molecule myeloid differentiation primary-response protein kinases 88 (MyD88), a shared adaptor protein of TLRs triggers the downstream pathways like NF-κB and MAPK cascades [56]. Among the MAPK, extracellular signal-regulated kinase 1 and 2 (ERK1/2), c-Jun N-terminal kinase (JNK) and p38 (also known as MAPK14) kinases have been intensively studied, from which JNK and p38 kinases show a higher responsiveness [57].

In human oral keratinocytes (HOKs) it was demonstrated that *P. gingivalis* LPS could activate both p38 and JNK pathways by inducing phosphorylation of IκBa and p65 transcription factors. These results indicate that induction of LPS binding protein (LBP) expression in HOKs by *P. gingivalis* LPS involves NF-κB and p38 MAPK signaling pathways[58]. Further members of the MAPK family are mitogen-activated protein kinase 4 (MEK4 or MAP2K4) and c-Jun NH2 terminal kinase 1 (JNK1 or MAPK8). After stimulation, activated TLR2 may initiate a cascade activation of MAPKs including MEK4 [59]. JNK1 is a downstream target of MEK4 [60]. In a human lung carcinoma type II epithelial cell line (A549) stimulation with LPS enhanced phosphorylation of MEK 4 and JNK1 in a time-dependent manner [61].

The results of our study demonstrate that *P. gingivalis* and its membrane fraction, induced RNA up-regulation of the NF-κB and p38 MAPK, MEK4-JNK1 signaling pathways. Furthermore, it was shown that a malignant oral epithelial cell line responded in a similar manner as non-transformed oral keratinocytes. These results are interesting since p38 MAPK and MEK4-JNK1 signaling pathways are known to be involved in tumor microenvironment and cancer growth control.

Head and neck squamous cell carcinoma (HNSCC) tissues express high levels of active p38 and the blockade of its signaling pathway caused significant inhibition of head and neck squamous cell carcinoma (HNSCC) proliferation [62]. Stromal fibroblasts of a variety of invasive malignant tumors express collagenase-1 (matrix metalloproteinase (MMP)-1), which was shown to correlate with the activation of c-Jun NH2-terminal kinase (JNK) and p38 mitogen-activated protein kinase and phosphorylation of c-Jun. It was also demonstrated that JNK2 is required for induction of fibroblast collagenase-3expression [63].

The data of the present study show a possible link between infection with *P. gingivalis* and oral squamous cell carcinomas, considering that periodontal disease has been associated with the risk for oral tumors [64].

Huynh et al. (2016) reported that in human oral epithelial cells interleukin regulation factor (IRF) 6 expression was strongly up-regulated upon challenge with *P. gingivalis*. IRF6 thus is acting downstream of IL-1 receptor (IL-1R)–associated kinase 1 to induce the expression of the IL-1 family cytokine IL-36 gamma responding to *P. gingivalis* [8]. The transcription factor IRF5 is a co-regulator of IFN-β [65] that exhibits a number of functions, including virus-mediated activation of interferon [66]. The results of the profiler array analysis showed up-regulation of IRF5 in SCC-25 cells by membrane fractions, as well as by whole bacteria (also in PHGK). These results were confirmed by quantitative real time PCR assays. These results suggest that IRFs presumably support inflammatory processes upon infection with *P. gingivalis*.

## Conclusions

In malignant and primary human oral epithelial cells, *P. gingivalis* and its membrane fraction induced up-regulation of a number of genes. These genes are involved in the downstream signaling pathway of the pro-inflammatory active transcription factor NF-κB and some members of the MAPK family. These kinases participate in the downstream signaling pathway for gene induction of pro-inflammatory cytokines and are involved in cancer proliferation and control.

## Additional Files

Additional file 1: Table S4. Ct values of up-regulation of genes in *P. gingivalis* membrane and whole bacteria treated SCC-25 cells. Ct values from qRT-PCR of NF-κB, IKBKB, MAP2K4, MAPK8, MAPK 14, IRF5, JUN, IRAK3 and TOLLIP in SCC-25 cells after 24 h stimulation with *P. gingivalis* membrane fraction = TM or *P. gingivalis* whole bacteria = WB, analyzed by ΔΔCt method, shown as absolute fold induction of RNA expression relative to non-stimulated samples, normalized to the house keeping gene GAPDH, n = 9, ‡ = *p* < 0.01. (DOCX 23 kb)
Additional file 2: Table S5. Ct values of up-regulation of genes in *P. gingivalis* membrane and whole bacteria treated PHGK cells. Ct values from qRT-PCR of NF-κB, IKBKB, MAP2K4, MAPK8, MAPK 14, IRF5, JUN, IRAK3 and TOLLIP in PHGK cells after 24 h stimulation with *P. gingivalis* membrane fraction = TM or *P. gingivalis* whole bacteria = WB, analyzed by ΔΔCt method, shown as absolute fold induction of RNA expression relative to non-stimulated samples, normalized to the house keeping gene GAPDH, n = 9, ‡ = *p* < 0.01. (DOCX 19 kb)

## Abbreviations

IRAK: Interleukin receptor-associated kinase
AG: Antigen
APCs: Antigen-presenting cells
DCs: Dendritic cells
ERK1/2: Extracellular signal-regulated kinase 1 and 2
*F. nucleatum*: *Fusobacterium nucleatum*
GECs: Gingival epithelial cells
HNSCC: Head and neck squamous cell carcinoma
HOKs: Human oral keratinocytes
IFN-γ: Interferon gamma
IG: Immunoglobulin
IκBα: Nuclear factor of kappa light polypeptide gene enhancer in B-cells inhibitor alpha
IL: Interleukin
ILT-3: Immunoglobulin-like transcript 3
IRF5/6: Interferon regulatory factor 5/6
JNK: c-Jun N-terminal kinase
LBP: LPS binding protein
LPS: Lipopolysaccharide
MAPK: Mitogen-activated protein kinase
MoDCs: Monocyte derived dendritic cells
NF-κB: Nuclear factor-kappaB
NOD: Nucleotide-binding oligomerization domain
*P. gingivalis*: *Porphyromonas gingivalis*
PAMPs: Pathogen-associated molecular patterns
PARs: Protease-activated receptors
PD-1: Programmed death-1
PD-L1: Programmed death receptor ligend 1
PHGK: Primary human gingival keratinocytes
PRRs: Pattern recognition receptors
QRT-PCR: Quantitative real time PCR
SCC-25 cells: Squamous cell carcinoma-25 cells
TCR: T-cell receptor
TLR: Toll-like receptor
TOLLIP: Toll-interacting protein
T_reg_: Regulatory T cells
VCAM 1: Vascular cell adhesion protein 1
