# Supplementary material for: Porphyromonas gingivalis activates NFκB and MAPK pathways in human oral epithelial cells
Source: BMC Immunol. 2017 Jan 5;18:1. doi: 10.1186/s12865-016-0185-5 (PMC5217430; doi:10.1186/s12865-016-0185-5)
Supplement: Additional file 1: Table S4. — Ct values of up-regulation of genes in P. gingivalis membrane and whole bacteria treated SCC-25 cells. Ct values from qRT-PCR of NF-κB, IKBKB, MAP2K4, MAPK8, MAPK 14, IRF5, JUN, IRAK3 and TOLLIP in SCC-25 cells after 24 h stimulation with P. gingivalis membrane fraction = TM or P. gingivalis whole bacteria = WB, analyzed by ΔΔCt method, shown as absolute fold induction of RNA expression relative to non-stimulated samples, normalized to the house keeping gene GAPDH, n = 9, ‡ = p < 0.01. (DOCX 23 kb) [file 12865_2016_185_MOESM1_ESM.docx]

| **SCC-25** | **Ct** | **Ct** | **Ct** | **Ct** | **Ct** | **Ct** | **Ct** | **Ct** | **Ct** | **Mean** | **St. Dev.** | **Δ Ct** | **ΔΔ Ct** | **2E-ΔΔ Ct** |
| --- | --- | --- | --- | --- | --- | --- | --- | --- | --- | --- | --- | --- | --- | --- |
| **GAPDH neg** | 30.57 | 29.73 | 30.45 | 31.51 | 31.41 | 31.61 | 31.55 | 31.50 | 31.20 | 31.06 | 0.66 |  |  |  |
| **NFκB neg** | 28.10 | 28.12 | 27.91 | 32.20 | 32.13 | 32.16 | 32.37 | 32.55 | 32.96 | 30.94 | 2.19 | -0.12 |  |  |
| **IκBκB neg** | 24.56 | 24.57 | 24.57 | 25.04 | 25.16 | 25.10 | 25.85 | 25.82 | 25.71 | 25.16 | 0.53 | -5.90 |  |  |
| **MAP2K4 neg** | 26.11 | 26.18 | 26.60 | 28.44 | 28.46 | 28.95 | 27.67 | 27.84 | 28.04 | 27.59 | 1.05 | -3.47 |  |  |
| **MAPK8 neg** | 23.72 | 23.22 | 22.92 | 25.34 | 24.75 | 24.17 | 24.78 | 24.25 | 24.18 | 24.15 | 0.77 | -6.91 |  |  |
| **MAPK14 neg** | 26.19 | 25.11 | 25.09 | 26.76 | 25.31 | 25.34 | 26.26 | 26.34 | 26.22 | 25.84 | 0.63 | -5.21 |  |  |
| **IRF5 neg** | 20.27 | 20.35 | 20.40 | 21.79 | 21.71 | 21.63 | 21.35 | 21.40 | 21.43 | 21.14 | 0.62 | -9.91 |  |  |
| **Jun neg** | 27.68 | 27.88 | 27.93 | 30.64 | 30.60 | 30.87 | 31.78 | 32.08 | 32.36 | 30.20 | 1.88 | -0.86 |  |  |
| **IRAK3 neg** | 24.92 | 24.69 | 24.50 | 26.79 | 26.62 | 26.40 | 26.67 | 26.14 | 26.26 | 25.89 | 0.92 | -5.17 |  |  |
| **Tollip neg** | 23.78 | 23.79 | 23.67 | 26.57 | 26.52 | 25.97 | 25.63 | 25.47 | 25.43 | 25.20 | 1.17 | -5.85 |  |  |
| **GAPDH TM** | 31.48 | 30.78 | 31.53 | 31.34 | 30.81 | 32.54 | 32.73 | 32.41 | 33.15 | 31.86 | 0.87 |  |  |  |
| **NFκB TM** | 28.77 | 28.91 | 28.70 | 30.30 | 30.51 | 30.30 | 29.86 | 29.76 | 29.92 | 29.67 | 0.70 | -2.19 | -2.08 | 4.22 |
| **IκBκB TM** | 23.48 | 23.47 | 23.42 | 24.09 | 24.11 | 24.04 | 23.70 | 23.70 | 23.61 | 23.74 | 0.28 | -8.13 | -2.22 | 4.67 |
| **MAP2K4 TM** | 26.22 | 26.15 | 26.23 | 26.64 | 26.74 | 26.94 | 25.48 | 25.54 | 25.68 | 26.18 | 0.53 | -5.68 | -2.21 | 4.61 |
| **MAPK8 TM** | 23.42 | 22.92 | 22.42 | 24.20 | 23.54 | 23.35 | 24.22 | 24.25 | 23.86 | 23.58 | 0,63 | -8.29 | -1.38 | 2.59 |
| **MAPK14 TM** | 24.62 | 24.82 | 24.69 | 24.61 | 24.73 | 24.57 | 24.34 | 24.43 | 24.34 | 24.57 | 0.17 | -7.29 | -2.07 | 4.21 |
| **IRF5 TM 24h** | 16.70 | 17.02 | 16.81 | 19.96 | 20.09 | 20.02 | 19.13 | 19.11 | 19.04 | 18.65 | 1.42 | -13.21 | -3.30 | 9.84 |
| **Jun TM** | 28.64 | 28.82 | 28.92 | 29.88 | 30.10 | 30.39 | 29.12 | 29.11 | 29.56 | 29.39 | 0.62 | -2.47 | -1.61 | 3.05 |
| **IRAK3 TM** | 25.12 | 24.65 | 24.53 | 26.38 | 25.84 | 25.98 | 24.83 | 24.34 | 24.28 | 25.12 | 0.78 | -6.76 | -1.58 | 3.00 |
| **Tollip TM** | 23.57 | 23.56 | 22.17 | 25.40 | 25.07 | 25.44 | 24.78 | 24.72 | 23.12 | 24.21 | 1.14 | -7.66 | -1.80 | 3.49 |
| **GAPDH WB** | 31.95 | 31.47 | 31.87 | 31.81 | 31.68 | 31.68 | 31.87 | 31.98 | 31.08 | 31.68 | 0.30 |  |  |  |
| **NFκB WB** | 30.69 | 30.99 | 31.36 | 32.08 | 31.89 | 32.12 | 29.38 | 29.84 | 30.07 | 30.94 | 1.01 | -0.74 | -0.63 | 1.55 |
| **IκBκB WB** | 24.26 | 24.18 | 23.89 | 24.70 | 24.53 | 24.31 | 25.19 | 25.12 | 24.76 | 24.55 | 0.44 | -7.13 | -1.23 | 2.34 |
| **MAP2K4 WB** | 26.89 | 27.06 | 26.76 | 27.48 | 27.83 | 27.83 | 25.87 | 26.31 | 25.87 | 26.88 | 0.76 | -4.80 | -1.33 | 2.52 |
| **MAPK8 WB** | 22.08 | 21.91 | 22.15 | 23.23 | 23.08 | 23.14 | 23.57 | 23.54 | 23.61 | 22.92 | 0.69 | -8.76 | -1.85 | 3.59 |
| **MAPK14 WB** | 24.81 | 24.93 | 25.00 | 24.57 | 24.64 | 24.70 | 25.08 | 25.08 | 25.23 | 24.89 | 0.23 | -6.79 | -1.57 | 2.98 |
| **IRF5 TM WB** | 19.12 | 19.05 | 18.68 | 21.19 | 20.87 | 20.66 | 19.56 | 19.32 | 19.10 | 19.73 | 0.92 | -11.95 | -2.04 | 4.12 |
| **Jun WB** | 30.16 | 29.98 | 29.84 | 31.13 | 31.05 | 30.74 | 29.41 | 29.15 | 29.06 | 30.06 | 0.78 | -1.62 | -0.77 | 1.70 |
| **IRAK3 WB** | 23.98 | 24.15 | 24.33 | 26.30 | 26.21 | 26.66 | 23.82 | 23.96 | 23.84 | 24.81 | 1.21 | -6.87 | -1.70 | 3.25 |
| **Tollip WB** | 24.06 | 24.07 | 22.76 | 26.48 | 26.53 | 26.37 | 24.19 | 24.11 | 22.93 | 24.61 | 1.48 | -7.07 | -1.22 | 2.32 |

**Tab. 4: Ct values of up-regulation of genes in *P. gingivalis* membrane and whole bacteria treated SCC-25 cells**

Ct values from qRT-PCR of NFκB, IKBKB, MAP2K4, MAPK8, MAPK 14, IRF5, JUN, IRAK3 and TOLLIP in SCC-25 cells after 24 h stimulation with *P. gingivalis* membrane fraction = TM or *P. gingivalis* whole bacteria = WB, analyzed by ΔΔCt method, shown as absolute fold induction of RNA expression relative to non-stimulated samples, normalized to the house keeping gene GAPDH, n = 9, ‡ = *p* < 0.01.
