# Supplementary material for: Porphyromonas gingivalis activates NFκB and MAPK pathways in human oral epithelial cells
Source: BMC Immunol. 2017 Jan 5;18:1. doi: 10.1186/s12865-016-0185-5 (PMC5217430; doi:10.1186/s12865-016-0185-5)
Supplement: Additional file 2: Table S5. — Ct values of up-regulation of genes in P. gingivalis membrane and whole bacteria treated PHGK cells. Ct values from qRT-PCR of NF-κB, IKBKB, MAP2K4, MAPK8, MAPK 14, IRF5, JUN, IRAK3 and TOLLIP in PHGK cells after 24 h stimulation with P. gingivalis membrane fraction = TM or P. gingivalis whole bacteria = WB, analyzed by ΔΔCt method, shown as absolute fold induction of RNA expression relative to non-stimulated samples, normalized to the house keeping gene GAPDH, n = 9, ‡ = p < 0.01. (DOCX 19 kb) [file 12865_2016_185_MOESM2_ESM.docx]

| **PHGK** | **Ct** | **Ct** | **Ct** | **Ct** | **Ct** | **Ct** | **Ct** | **Ct** | **Ct** | **Mean** | **St. Dev.** | **Δ Ct** | **ΔΔ Ct** | **2E-ΔΔ Ct** |
| --- | --- | --- | --- | --- | --- | --- | --- | --- | --- | --- | --- | --- | --- | --- |
| **GAPDH neg** | 33.74 | 33.80 | 33.26 | 29.37 | 29.26 | 29.27 | 33.55 | 33.19 | 32.60 | 32.00 | 2.06 |  |  |  |
| **NFκB neg** | 32.88 | 32.77 | 32.84 | 27.34 | 27.33 | 27.38 | 32.95 | 33.03 | 32.87 | 31.04 | 2.77 | -0.96 |  |  |
| **IκBκB neg** | 26.15 | 26.20 | 26.15 | 23.50 | 23.36 | 23.41 | 24.93 | 24.67 | 24.62 | 24.78 | 1.19 | -7.23 |  |  |
| **MAP2K4 neg** | 29.05 | 29.40 | 30.37 | 26.24 | 26.39 | 26.93 | 28.28 | 28.42 | 29.09 | 28.24 | 1.43 | -3.76 |  |  |
| **MAPK8 neg** | 25.83 | 25.14 | 24.72 | 22.69 | 22.22 | 21.76 | 24.99 | 24.23 | 23.73 | 23.92 | 1.42 | -8.08 |  |  |
| **MAPK14 neg** | 28.43 | 27.15 | 27.21 | 25.07 | 24.10 | 24.13 | 25.91 | 24.79 | 24.86 | 25.74 | 1.53 | -6,27 |  |  |
| **IRF5 neg** | 21.08 | 21.14 | 21.04 | 18.87 | 18.94 | 18.93 | 19.02 | 21.13 | 21.68 | 20.20 | 1.21 | -10.6 |  |  |
| **Jun neg** | 31.09 | 31.29 | 31.33 | 31.70 | 31.79 | 31.99 | 31.70 | 31.79 | 31.99 | 31.63 | 0.32 | -0.81 |  |  |
| **IRAK3 neg** | 27.04 | 26.71 | 26.54 | 24.09 | 23.86 | 23.65 | 24.86 | 24.56 | 24.44 | 25.08 | 1.32 | -5.74 |  |  |
| **Tollip neg** | 25.77 | 25.72 | 25.93 | 24.05 | 24.05 | 23.96 | 23.70 | 23.62 | 23.51 | 24.48 | 1.02 | -6,34 |  |  |
| **GAPDH TM** | 32.34 | 32.43 | 32.48 | 30.58 | 30.47 | 30.48 | 34.54 | 34.66 | 32.11 | 32.23 | 1.59 |  |  |  |
| **NFκB TM** | 31.20 | 31.65 | 32.10 | 28.83 | 29.17 | 29.30 | 31.46 | 31.46 | 31.77 | 30.77 | 1.28 | -1.46 | -0.50 | 1.41 |
| **IκBκB TM** | 24,59 | 24,31 | 24,06 | 23,58 | 23,38 | 23,17 | 21,68 | 23,07 | 22,69 | 23.39 | 0.89 | -8.84 | -1.61 | 3.05 |
| **MAP2K4 TM** | 28.44 | 28.77 | 28.52 | 26.63 | 27.09 | 26.75 | 23.85 | 24.24 | 23.82 | 26.46 | 2.02 | -5.77 | -2.01 | 4.03 |
| **MAPK8 TM** | 24.02 | 23.84 | 23.87 | 22.92 | 22.69 | 22.78 | 23.39 | 23.11 | 23.37 | 23.33 | 0.49 | -8.90 | -0.82 | 1.76 |
| **MAPK14 TM** | 24.38 | 24.41 | 24.47 | 24.30 | 24.31 | 24.44 | 24.38 | 24.41 | 24.47 | 24.39 | 0.06 | -7.83 | -1.57 | 2.96 |
| **IRF5 TM 24h** | 19.04 | 18.49 | 18.27 | 18.35 | 18.41 | 18.64 | 18.75 | 19.28 | 19.55 | 20.75 | 0.44 | -12.2 | -1.57 | 2.96 |
| **Jun TM** | 30.52 | 30.23 | 30.47 | 31.35 | 30.68 | 30.93 | 31.35 | 30.68 | 30.93 | 30.79 | 0.38 | -2.14 | -2.95 | 7.73 |
| **IRAK3 TM** | 25.56 | 25.63 | 26.06 | 23.59 | 23.67 | 24.06 | 24.09 | 24.13 | 24.39 | 24.58 | 0.92 | -8.36 | -2.62 | 6.14 |
| **Tollip TM** | 25.54 | 25.49 | 25.69 | 23.94 | 23.90 | 23.21 | 23.69 | 23.70 | 24.16 | 24.37 | 0.94 | -8.57 | -2.22 | 4.67 |

**Tab. 5: Ct values of up-regulation of genes in *P. gingivalis* membrane and whole bacteria treated PHGK cells**

Ct values from qRT-PCR of NFκB, IKBKB, MAP2K4, MAPK8, MAPK 14, IRF5, JUN, IRAK3 and TOLLIP in PHGK cells after 24 h stimulation with *P. gingivalis* membrane fraction = TM or *P. gingivalis* whole bacteria = WB, analyzed by ΔΔCt method, shown as absolute fold induction of RNA expression relative to non-stimulated samples, normalized to the house keeping gene GAPDH, n = 9, ‡ = *p* < 0.01.
